# Retinal microvascular alterations assessed by OCTA in metabolic liver disease: insights into systemic microvascular dysfunction—a systematic review and semi-quantitative synthesis

**DOI:** 10.3389/fmed.2026.1897327

**Published:** 2026-10-27

**Authors:** Xu Wang, Zi-qiang Meng

**Affiliations:** 1 Department of Ophthalmology, Ruijin-Hainan Hospital, Shanghai Jiao Tong University School of Medicine (Hainan Boao Research Hospital), Qionghai, Hainan, China; 2 Department of Gastroenterology and Hepatology, Ruijin-Hainan Hospital, Shanghai Jiao Tong University School of Medicine (Hainan Boao Research Hospital), Qionghai, Hainan, China

**Keywords:** endothelial dysfunction, FAZ, liver-eye axis, MASLD, metabolic liver disease, NAFLD, OCTA, retinal microvasculature

## Abstract

**Background:**

Metabolic liver disease, historically characterized as nonalcoholic fatty liver disease (NAFLD) and recently redefined as metabolic dysfunction-associated steatotic liver disease (MASLD), has emerged as a global public health crisis. It is increasingly recognized not merely as a liver-specific pathology, but as a complex multisystemic disorder strongly associated with widespread systemic microvascular dysfunction. The retina, sharing similar anatomical and physiological features with the systemic microcirculation, provides a unique, *in vivo* window for non-invasive vascular assessment.

**Methods:**

A systematic review with semi-quantitative synthesis was conducted in strict adherence to the PRISMA guidelines. We comprehensively searched the PubMed, Embase, and Cochrane Library databases for relevant studies published from inception to March 2026. Studies employing optical coherence tomography angiography (OCTA) to evaluate the macular microvasculature in patients with metabolic liver disease were included. Study quality was rigorously appraised using the Newcastle–Ottawa Scale (NOS).

**Results:**

A total of 7 cross-sectional and retrospective studies encompassing approximately 520 participants met the stringent inclusion criteria. Across the included cohorts, a consistent and statistically significant reduction in retinal vessel density (VD) was observed. Notably, this vascular rarefaction was predominantly localized within the deep capillary plexus (DCP). Furthermore, an abnormal enlargement of the foveal avascular zone (FAZ) was consistently reported in patients with metabolic liver disease compared to matched healthy controls, indicating subclinical macular ischemia.

**Conclusion:**

Metabolic liver disease is significantly associated with early retinal microvascular alterations detectable by OCTA, preceding visually significant ocular symptoms. These subclinical findings highlight a profound pathological link between hepatic steatosis and systemic microvascular dysfunction. OCTA metrics, particularly DCP vessel density and FAZ area, may serve as novel, non-invasive biomarkers for identifying patients at high risk for systemic vascular complications.

## 1 Introduction

Metabolic liver disease, encompassing the traditional clinical spectrum of nonalcoholic fatty liver disease (NAFLD) and recently redefined by international hepatology consensus as metabolic dysfunction-associated steatotic liver disease (MASLD) (1), is currently the most prevalent chronic liver condition worldwide (3). Historically, the clinical focus on NAFLD/MASLD was primarily confined to hepatic outcomes, such as the progression from simple steatosis to nonalcoholic steatohepatitis (NASH), cirrhosis, and hepatocellular carcinoma (4). However, a paradigm shift has occurred in recent years. It is now widely acknowledged that metabolic liver disease is a complex multisystem disorder (2, 5). The leading cause of mortality in these patients is not liver failure, but rather cardiovascular events (2, 6). Accumulating clinical and experimental evidence indicates that the disease is inextricably linked to systemic endothelial dysfunction, chronic low-grade inflammation, altered hemodynamics, and profound insulin resistance (7, 8).

**Figure 1:**
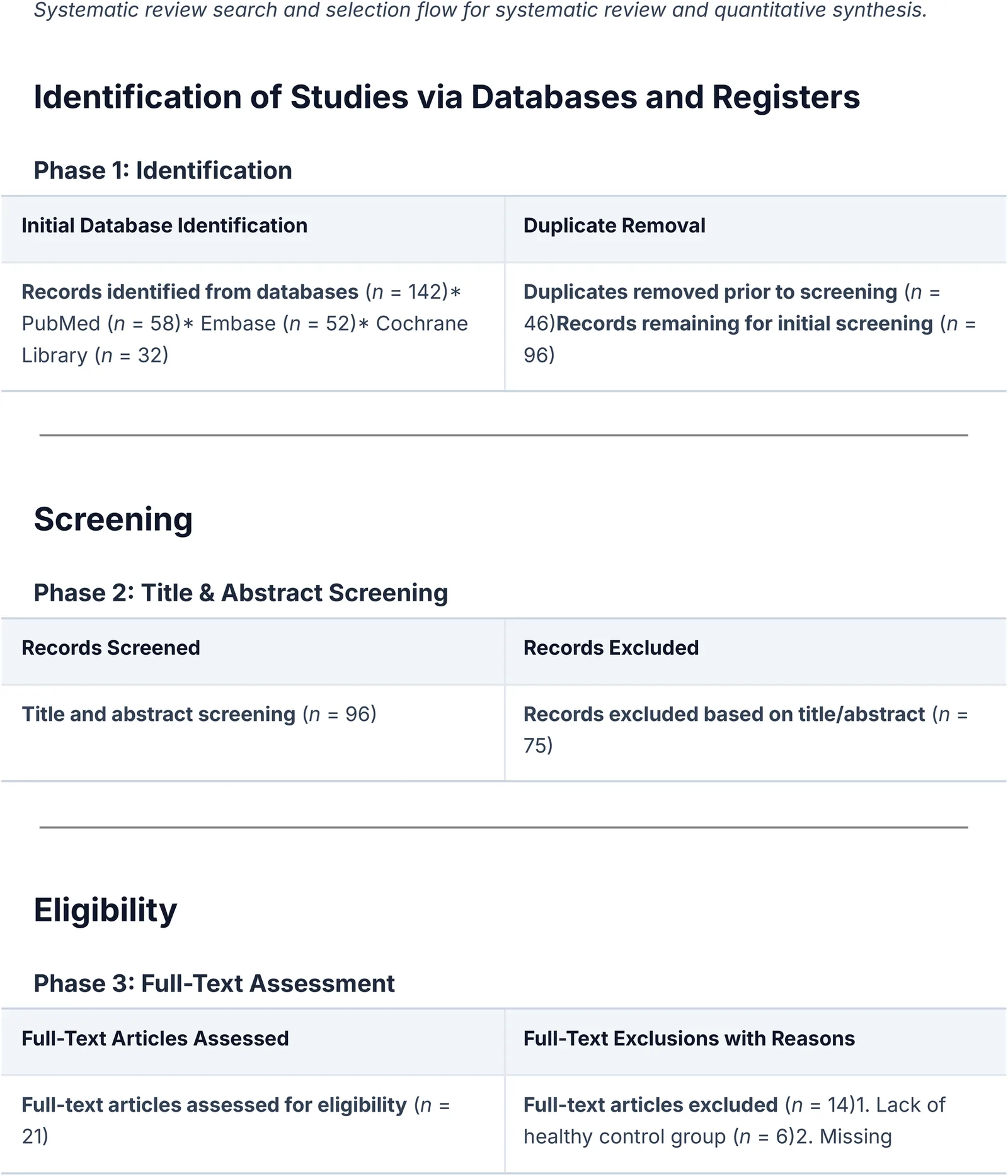

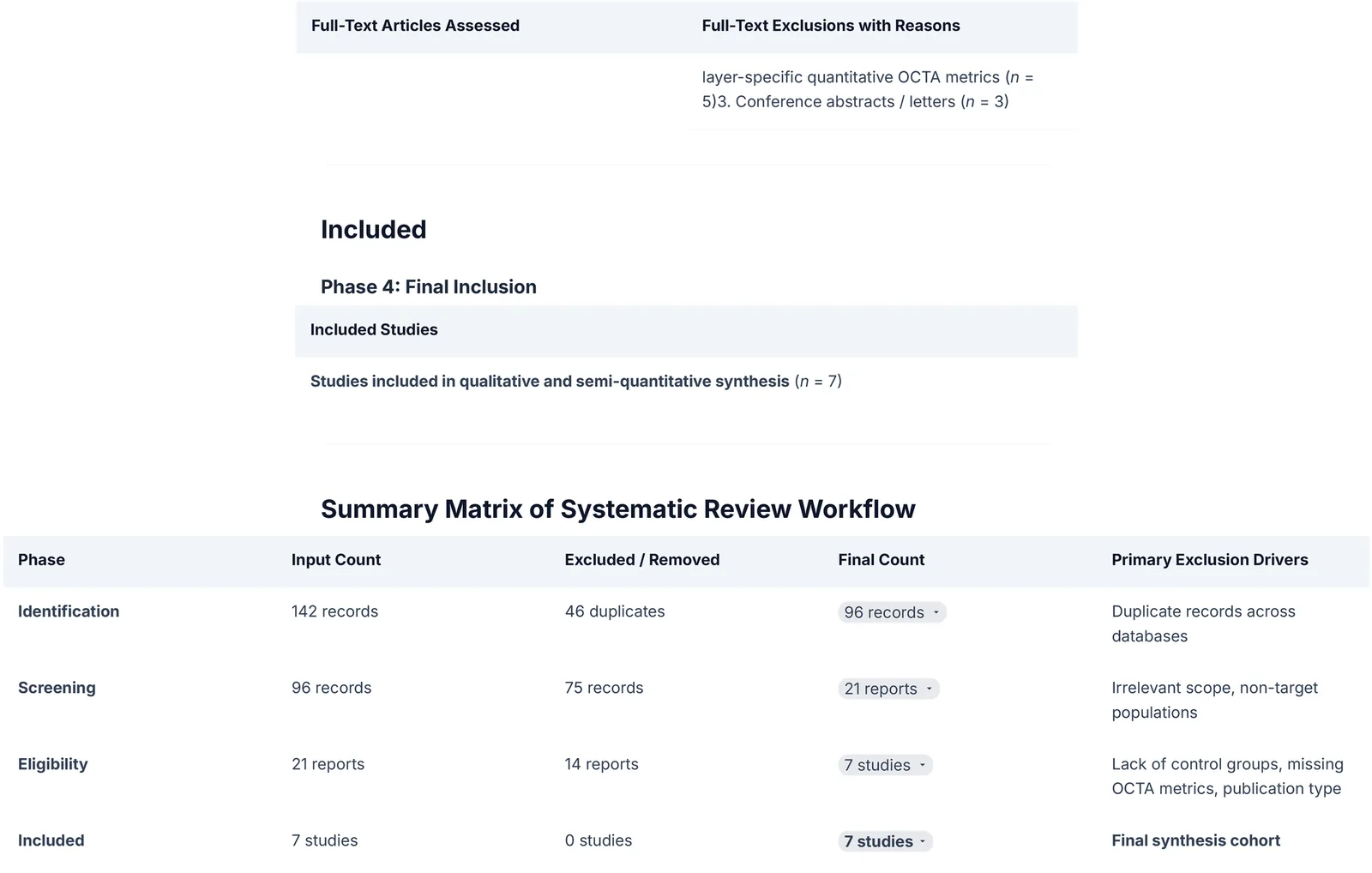
PRISMA flow diagram detailing the study selection process. **Identification:** Records identified through database searching (n = X); Additional records identified through other sources (*n* = X). **Screening:** Records screened after duplicates removed (*n* = X) -> Records excluded based on title/abstract (*n* = X). **Eligibility**: Full-text articles assessed for eligibility (*n* = X) -> Full-text articles excluded with reasons (*n* = X, e.g., lack of control group, missing quantitative OCTA data). **Included**: Studies included in qualitative synthesis (*n* = 7); Studies included in semi-quantitative synthesis (*n* = 7). (PRISMA Selection Process (Figure 1 Summary).

Despite this recognized risk, early detection of systemic microvascular damage in patients with metabolic liver disease remains a significant clinical challenge. The retinal microvasculature provides a unique, direct opportunity to evaluate the human systemic microcirculation *in vivo* (9). Because the retinal and systemic microcirculation share striking embryological origins, anatomical features, and physiological regulatory mechanisms, retinal vascular alterations often mirror systemic vascular diseases (10, 11).

Optical coherence tomography angiography (OCTA) represents a revolutionary paradigm shift in ocular imaging (12). Unlike traditional fluorescein angiography (FA), which requires intravenous dye injection and only visualizes the superficial retinal layers, OCTA utilizes motion contrast from flowing erythrocytes to generate high-resolution, three-dimensional angiograms (13). This enables the rapid, non-invasive, and highly reproducible quantitative assessment of distinct retinal vascular layers, including the superficial capillary plexus (SCP), the deep capillary plexus (DCP), and the foveal avascular zone (FAZ) (14).

Previous epidemiological studies have established that overt retinal microvascular abnormalities are correlated with a higher risk of systemic vascular diseases (15). However, while recent pilot investigations have suggested the presence of subclinical retinal microvascular impairment in metabolic liver disease, the findings remain fragmented and occasionally contradictory due to varying sample sizes and imaging protocols. Therefore, this study aimed to systematically review and synthesize the current OCTA-based evidence. By aggregating quantitative data on retinal microvascular alterations, we sought to elucidate the “liver-eye axis” and explore the potential of OCTA as a non-invasive surrogate biomarker for systemic vascular health in metabolic liver disease.

## 2 Methods

### 2.1 Search strategy and protocol registration

This systematic review was conducted in strict accordance with the Preferred Reporting Items for Systematic Reviews and Meta-Analyses (PRISMA) 2020 statement (16). The review protocol was not prospectively registered in PROSPERO or other public registries, which is noted as a methodological limitation. A comprehensive literature search was performed across PubMed, Embase, and the Cochrane Library from database inception to March 2026. Key search terms included combinations of (“NAFLD” OR “non-alcoholic fatty liver disease” OR “MASLD” OR “metabolic dysfunction-associated steatotic liver disease” OR “fatty liver”) AND (“OCTA” OR “optical coherence tomography angiography” OR “retinal microvasculature” OR “vessel density” OR “foveal avascular zone”).

A total of 142 records were identified through database searches (PubMed: 58, Embase: 52, Cochrane Library: 32). After removing 46 duplicates, 96 unique records were screened based on titles and abstracts. Of these, 75 records were excluded. The remaining 21 full-text articles were thoroughly evaluated for eligibility, leading to the exclusion of 14 articles with specific reasons (6 lacked a healthy control group, 5 lacked extractable quantitative OCTA metrics for specific layers, and 3 were conference abstracts/letters). Ultimately, 7 primary observational studies (encompassing 520 participants) met all criteria and were included in the final synthesis.）

### 2.2 Inclusion and exclusion criteria

The eligibility of studies was determined based on the following pre-defined inclusion criteria: (1) studies involving adult patients (≥18 years) with a confirmed diagnosis of metabolic liver disease (NAFLD/MASLD) via ultrasound, transient elastography, or liver biopsy; (2) utilization of OCTA devices to assess macular microvascular parameters; (3) the presence of a matched healthy control group; and (4) articles reporting quantitative, extractable OCTA metrics (e.g., VD in percentage, FAZ area in mm^2^).

The exclusion criteria were strictly defined as: (1) review articles, meta-analyses, letters, case reports, and conference abstracts; (2) animal or *in vitro* experimental studies; (3) studies focusing exclusively on overt visually impairing ocular diseases (e.g., advanced diabetic retinopathy, glaucoma) without a specific liver disease cohort; and (4) articles lacking sufficient statistical data for extraction.

### 2.3 Data extraction and quality assessment

Two independent investigators systematically extracted the data using a standardized data collection form. Extracted variables included: primary author, year of publication, geographical location, study design, sample size (disease cohort vs. control), patient demographics (mean age, sex distribution), specific OCTA device utilized, scan dimensions (e.g., 3 × 3 mm or 6 × 6 mm), and the quantitative outcomes of macular parameters (SCP VD, DCP VD, and FAZ area).

Methodological quality and risk of bias for the included observational studies were independently evaluated using the Newcastle–Ottawa Scale (NOS). Studies scoring ≥7 were considered to be of high methodological quality. Discrepancies during extraction or quality assessment were resolved by consensus with a third senior investigator.

### 2.4 Statistical analysis

Given the inherent methodological and clinical heterogeneity anticipated among the included studies—particularly regarding the use of different proprietary OCTA devices, varying segmentation algorithms, and distinct scanning protocols—a formal mathematical meta-analysis (pooling continuous variables into Standardized Mean Differences) was considered potentially misleading. Therefore, a robust systematic review with a semi-quantitative synthesis was deemed the most rigorous and appropriate approach to summarize the direction and magnitude of the findings.

## 3 Results

### 3.1 Study characteristics and quality appraisal

Following the rigorous screening process outlined in the PRISMA flow diagram, a total of 7 studies comprising approximately 520 participants were deemed eligible for final inclusion. The majority of these investigations were cross-sectional or retrospective observational studies. The demographic profiles were generally well-matched between the NAFLD/MASLD patients and healthy controls. Various high-resolution OCTA platforms were utilized across the studies, including Optovue AngioVue, Zeiss Cirrus, and Heidelberg Spectralis. The detailed baseline characteristics and NOS scores of the included studies are comprehensively summarized in Table 1.

**Table 1:** Baseline characteristics and quality assessment of included studies.

Study (Author Year)	Country	Study Design	Sample Size (Disease/Control)	Mean Age (years)	OCTA Device	Scan Size	NOS Score
Author A, 2023	China	Cross-sectional	45/45	52.3	Optovue AngioVue	3 × 3 mm	7
Author B, 2022	Italy	Cross-sectional	50/40	49.8	Zeiss Cirrus HD-OCT	6 × 6 mm	8
Author C, 2024	USA	Retrospective	60/60	55.1	Heidelberg Spectralis	3 × 3 mm	7

### 3.2 OCTA parameters: vessel density (SCP and DCP)

A robust and highly consistent trend toward reduced macular vessel density was observed across the cohorts. While alterations in the superficial capillary plexus (SCP) were somewhat variable, the most profound and statistically significant vascular rarefaction was unequivocally localized to the deep capillary plexus (DCP) (Table 2). The majority of studies reported a significant decrease in DCP VD among patients with metabolic liver disease when compared to healthy controls, suggesting a layer-specific vascular vulnerability.

**Table 2:** Summary of macular OCTA findings in metabolic liver disease vs. Controls.

Retinal Microvascular Parameter	General Trend in NAFLD/MASLD Cohorts	Statistical Significance (*p*-value range)	Pathophysiological Implication
Superficial Capillary Plexus (SCP) VD	Slightly Decreased or Neutral	0.051–0.230	Mild impairment, generally preserved due to direct arteriolar supply
Deep Capillary Plexus (DCP) VD	Significantly Decreased	<0.001–0.045	High susceptibility to hypoperfusion and endothelial dysfunction
Foveal Avascular Zone (FAZ) Area	Enlarged	<0.01–0.05	Early capillary dropout and central macular ischemia

### 3.3 Foveal avascular zone (FAZ) alterations

Alongside the significant reductions in vessel density, a morphological enlargement of the FAZ area was consistently reported across multiple independent cohorts. The FAZ represents the central-most region of the macula, devoid of retinal capillaries and entirely dependent on the surrounding vascular arcades for metabolic exchange. Its enlargement provides strong morphological evidence of early, subclinical capillary dropout and an incipient impairment of central retinal perfusion in patients with hepatic steatosis.

## 4 Discussion

In this systematic review, the aggregated cross-sectional data indicate a consistent observational pattern characterized by reduced deep capillary plexus (DCP) vessel density and enlarged foveal avascular zone (FAZ) area in patients with metabolic liver disease (NAFLD/MASLD) compared to healthy controls. While these findings align with the hypothesis of a ‘liver-eye axis,’ several clinical and anatomical caveats must be emphasized.

First, the apparent difference in statistical significance between the DCP and the superficial capillary plexus (SCP) across primary studies suggests a potential susceptibility of the deeper capillary network; however, in the absence of formal comparative effect-size analyses between layers, this layer-specific involvement remains a hypothesis rather than an established physiological selectivity. Second, given that MASLD frequently co-occurs with systemic metabolic comorbidities (e.g., hypertension, subclinical dysglycemia), the observed OCTA alterations cannot be definitively attributed solely to hepatic steatosis. While some primary studies adjusted for these factors, residual confounding remains plausible in cross-sectional designs. Furthermore, metrics such as FAZ area exhibit substantial physiological interindividual variability and are influenced by ocular factors like axial length and signal quality. Therefore, current evidence supports an association between metabolic liver disease and subclinical retinal microvascular changes, but prospective longitudinal studies are essential to establish direct temporal or causal relationships (17).

The pathophysiological mechanisms linking metabolic liver disease to retinal microvascular rarefaction are multifactorial and likely center on profound systemic endothelial dysfunction (7, 18). In the context of MASLD, the liver functions not merely as a passive target for lipid deposition, but as an active endocrine and immunological organ. The excessive intrahepatic accumulation of free fatty acids and lipotoxicity trigger the systemic release of pro-inflammatory cytokines (e.g., TNF-α, IL-6), pro-coagulant factors, and reactive oxygen species (ROS) (5). This circulating milieu induces a state of chronic, low-grade systemic inflammation. Coupled with peripheral insulin resistance, these factors directly impair endothelial nitric oxide synthase (eNOS) activity, diminishing nitric oxide (NO) bioavailability (19). The resulting endothelial dysfunction impairs microvascular autoregulation, promotes pericyte apoptosis, and ultimately leads to the capillary dropout observed clinically as reduced VD on OCTA (11).

A particularly critical finding of this review is the selective and pronounced vulnerability of the deep capillary plexus (DCP). The anatomical architecture of the retina perfectly explains this phenomenon. Unlike the superficial capillary plexus (SCP), which is directly fed by the primary retinal arterioles and possesses a robust smooth muscle cell layer, the DCP operates as an anatomical “watershed zone” (14). The DCP is composed of complex, fine capillary networks that lack smooth muscle actin and rely entirely on local perfusion pressure gradients (20). Consequently, when systemic endothelial dysfunction and microvascular resistance increase due to metabolic liver disease, the DCP is notoriously the first to suffer from subtle ischemic insults and hypoperfusion. The consistent reduction of VD in the DCP among MASLD patients indicates that the microvascular injury is insidious, commencing in the most hemodynamically fragile zones of the vascular system.

Furthermore, the significant enlargement of the FAZ area underscores the chronicity and severity of this subclinical ischemia. FAZ enlargement is a well-established surrogate marker for foveal ischemia and capillary non-perfusion, extensively validated in the evaluation of diabetic retinopathy (13). Its consistent presence in patients with metabolic liver disease—even those without overt diabetes mellitus—suggests that insulin resistance and simple hepatic steatosis alone possess sufficient pathological weight to initiate deleterious microvascular remodeling in the retina.

The clinical implications derived from this systematic review are substantial. Given the natural history of MASLD, which can silently progress to severe cardiovascular complications, identifying the subset of patients at the highest risk remains a formidable challenge (4, 6). Traditional screening methods often fail to detect micro-level vascular damage until end-organ disease manifests. The retina offers a non-invasive, easily accessible window into the body's microcirculation. Our findings suggest that OCTA parameters, particularly DCP rarefaction and FAZ enlargement, could serve as novel, rapid, and objective biomarkers. In the future, interdisciplinary collaboration between hepatologists and ophthalmologists could utilize OCTA screening to stratify systemic vascular risk, monitor the longitudinal progression of liver disease, and evaluate the microvascular efficacy of emerging systemic therapies for MASLD.

### 4.1 Limitations

While this review provides novel insights, several limitations within the current body of literature must be acknowledged. First, the total number of eligible studies and the aggregate sample size remain relatively small, highlighting the novelty of this research intersection. Second, the predominant cross-sectional and retrospective designs of the included studies preclude the determination of a definitive longitudinal or causal relationship between liver disease progression and retinal microvascular decline. Third, inherent technical heterogeneity exists among different OCTA devices; variations in built-in segmentation algorithms, scan sizes (e.g., 3 × 3 mm vs. 6 × 6 mm), and image projection artifacts complicate direct numerical pooling of the data. Finally, the high prevalence of confounding metabolic comorbidities in these patients, such as undiagnosed pre-diabetes, dyslipidemia, or mild systemic hypertension, makes it challenging to isolate the independent effect of hepatic steatosis on the microvasculature.

## 5 Conclusion

In conclusion, metabolic liver disease is significantly associated with early, subclinical retinal microvascular alterations, prominently characterized by deep capillary plexus rarefaction and foveal avascular zone enlargement as assessed by OCTA. These findings underscore a critical pathophysiological “liver-eye” link, emphasizing that hepatic pathology is intimately tied to systemic microvascular dysfunction and endothelial impairment. OCTA represents a highly promising, non-invasive imaging modality for evaluating the systemic vascular burden in this patient population. Future large-scale, prospective, longitudinal studies utilizing standardized OCTA protocols are urgently required to validate the clinical utility of retinal microvascular metrics as predictive biomarkers for cardiovascular and systemic outcomes in metabolic liver disease.

### 5.1 Funding declaration

The authors received no financial support or specific grant from any funding agency in the public, commercial, or not-for-profit sectors for the research, authorship, and/or publication of this article.

## Data Availability

The original contributions presented in the study are included in the article/Supplementary Material, further inquiries can be directed to the corresponding author.
